# Functional near‐infrared spectroscopy detects brain changes for apathy and pain in patients with Alzheimer's disease and related dementias

**DOI:** 10.1002/alz70856_098911

**Published:** 2025-12-24

**Authors:** Allison J. Huff, Juyoung Park, Samuel Montero‐Hernandez, Lindsey Park, Chiyoung Lee, Luca Pollonini, Hyochol Ahn

**Affiliations:** ^1^ University of Arizona, Tucson, AZ, USA; ^2^ University of Arizona College of Nursing, Tucson, AZ, USA; ^3^ University of Birmingham, Birmingham, Birmingham, United Kingdom; ^4^ University of Houston, Houston, TX, USA

## Abstract

**Background:**

Alzheimer's Disease and Related Dementias (ADRD) are degenerative and progressive in nature and are often accompanied by chronic pain and neuropsychiatric symptoms, which can be early signs and aggravators of ADRD. This study explores the relationship between self‐reported pain, neuropsychiatric symptoms, and pain‐evoked cortical hemodynamic changes using functional near infrared spectroscopy (fNIRS) in the prefrontal and motor brain cortices, stratified by high or low cognitive function in individuals with ADRD.

**Method:**

Cerebral hemodynamic changes in five brain regions of interest were measured using fNIRS from participants (*n* = 40) with ADRD and chronic pain. Self‐reported pain was measured using Numeric Rating System, and self‐reported depression and apathy were measured using NeuroPsychiatric Inventory. High and low cognitive function groups were determined based on scores from the Mini‐Mental Status Exam and Telephone‐Montreal Cognitive Assessment completed at baseline. We investigated the relationships of thermal pain‐evoked cerebral hemodynamic changes in each region of interest and self‐reported pain and self‐reported depression and apathy stratified by high or low cognitive function.

**Results:**

The study revealed significant negative correlations for oxy‐hemoglogin and apathy in the right frontal region associated with low cognitive function (*p* = .04) and significant positive correlations for oxy‐hemoglobin and apathy in the right motor region (*p* = .04) and for oxy‐hemoglobin and pain in the middle frontal region (*p* = .04) associated with higher cognitive function.

**Conclusion:**

Study findings suggest that fNIRS may provide valuable biomarkers for apathy and depression in ADRD, with differential patterns based on cognitive function, suggesting neuropsychiatric symptoms may manifest differently depending on the patient's cognitive status. This could help clinicians better understand the severity and nature of neuropsychiatric symptoms and tailor interventions more effectively. Future studies should explore its utility in larger, diverse samples and clinical interventions targeting neuropsychiatric symptoms.

